# Excited States and Their Dynamics in CdSe Quantum Dots Studied by Two-Color 2D Spectroscopy

**DOI:** 10.1021/acs.jpclett.1c04110

**Published:** 2022-01-28

**Authors:** Zhengjun Wang, Nils Lenngren, Edoardo Amarotti, Albin Hedse, Karel Žídek, Kaibo Zheng, Donatas Zigmantas, Tõnu Pullerits

**Affiliations:** † Division of Chemical Physics and NanoLund, Lund University, P.O. Box 124, 22100 Lund, Sweden; ‡ ELI Beamlines, Institute of Physics, Czech Academy of Sciences, v.v.i., Za Radnicí 835, 252 41 Dolní Břežany, Czech Republic; § Regional Center for Special Optics and Optoelectronic Systems (TOPTEC), Institute of Plasma Physics of the Czech Academy of Sciences, 270 00 Prague 8, Czech Republic; ∥ Department of Chemistry, Technical University of Denmark, DK-2800 Kongens Lyngby, Denmark

## Abstract

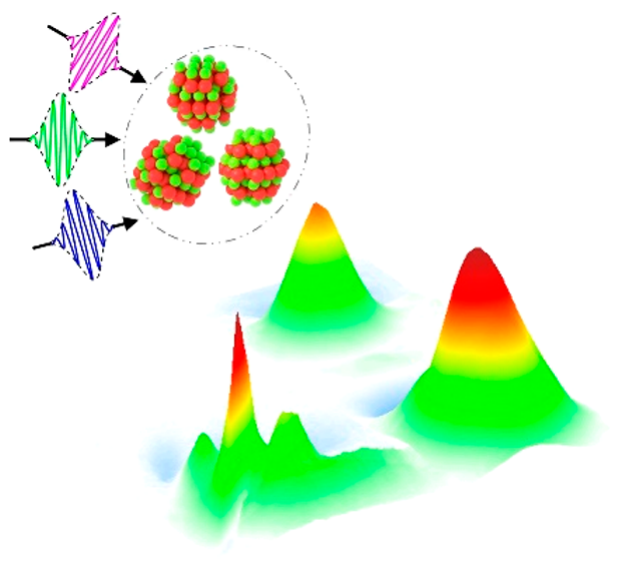

Quantum dots (QDs) form a promising family of nanomaterials for various applications in optoelectronics. Understanding the details of the excited-state dynamics in QDs is vital for optimizing their function. We apply two-color 2D electronic spectroscopy to investigate CdSe QDs at 77 K within a broad spectral range. Analysis of the electronic dynamics during the population time allows us to identify the details of the excitation pathways. The initially excited high-energy electrons relax with the time constant of 100 fs. Simultaneously, the states at the band edge rise within 700 fs. Remarkably, the excited-state absorption is rising with a very similar time constant of 700 fs. This makes us reconsider the earlier interpretation of the excited-state absorption as the signature of a long-lived trap state. Instead, we propose that this signal originates from the excitation of the electrons that have arrived in the conduction-band edge.

The discovery of the quantum size effect in colloidal semiconductor nanocrystals,^1,2^ the so-called quantum dots (QDs), opened a new topic in nanomaterial research. An important milestone of the following development was the introduction of the hot-injection method, enabling easy synthesis of high-quality monodisperse QDs.^3^ Since then, the field has been expanding toward a broad combination of materials, sophisticated structures, and optoelectronic devices,^4^ such as light-emitting diodes^5^ and microspectrometers.^6^ Throughout the years, numerous studies have addressed a broad set of fundamental questions regarding excited states and their dynamics in QDs.^7−12^ Several recent studies have addressed issues like high-intensity effects^13−16^ and the influence of charging on excited-state dynamics^17,14^—all important from the point of view of possible optoelectronic applications of QDs.

Recent developments in coherent multidimensional spectroscopy (CMDS)^18−27^ have opened new possibilities for investigating excited-state dynamics with a very high level of precision in both time and spectral resolution.^28,29^ The method has demonstrated its capabilities in studies of dynamics, including coherent evolution of both vibrational and electronic origin in both biomaterials and semiconductors.^30,31^ It has become increasingly popular to investigate quantum coherence, relaxation, and coupling of excited states by using CMDS.^28,30,32,33^ Numerous studies on excited-state dynamics in QDs have applied coherent 2D spectroscopy.^18,23,28,33,34^

In this Letter, we extend our previous 2D spectroscopy study of CdSe QDs at 77 K^23^ by significantly lengthening the spectral coverage (now ranging from 15200 to 21300 cm^–1^) via applying additional pulse energies and thereby using two-color 2D spectroscopy.

The peaks of the 2D spectra originate from many different optical responses, such as ground-state bleach (GSB), stimulated emission (SE), and excited-state absorption (ESA).^35,36^ Furthermore, depending on the pulse ordering, we can distinguish the rephasing and nonrephasing signals, which provide complementary information. Using the breadth of available information, we obtain a detailed description of an extensive range of the CdSe QD states and their excitation dynamics during the first 1400 fs after excitation.

2D electronic spectroscopy^37^ (2DES) is a third-order nonlinear optical method which uses three short laser pulses acting on the sample. Between these three pulses, there are two delay times in the 2DES experiment. However, since the coherently generated signal also provides an instance of field–matter interaction, in total we have three time intervals to count, namely coherence time *t*_1_ (often noted as τ, between the first and the second pulses), population time *t*_2_ (often noted *T*, between the second and third pulses), and detection time *t*_3_ (also called *t*, between the third pulse and the signal generation).

In traditional 2D spectroscopy, the three pulses have the same carrier frequency, and consequently, the measurable energy region is the same in both spectral dimensions of the 2D map. In this work, we investigate the dynamics of the excited states of CdSe QDs by using pulses of different frequency, thereby broadening the spectral coverage of the experiment. This spectroscopic technique is called two-color 2D spectroscopy.^38,39^ In our two-color 2D spectroscopy measurement, the first two laser pulses (also called pump pulses) resonate with a high-energy state of the sample system, and the third laser pulse (also called the probe pulse) together with a local oscillator (LO) covers low-energy region near the band edge of the sample (for detailed experimental information, see the Supporting Information). That is the reason the ω_1_-axis and the ω_3_-axis are different in the two-color 2D spectrum shown in panels B_1_ and B_2_ of Figure 1. The spectrogram reveals a rich network of cross peaks in the two-color 2D spectrum. Two-color 2D spectroscopy shows more nondegenerate or off-diagonal traits, which provides knowledge on energy or coherence transitions between electronic levels.^38,39^ These peak networks reflect correlations between the excited states, and their population time dependence provides information about the dynamics among excited states in the CdSe QD system.

**Figure 1:**
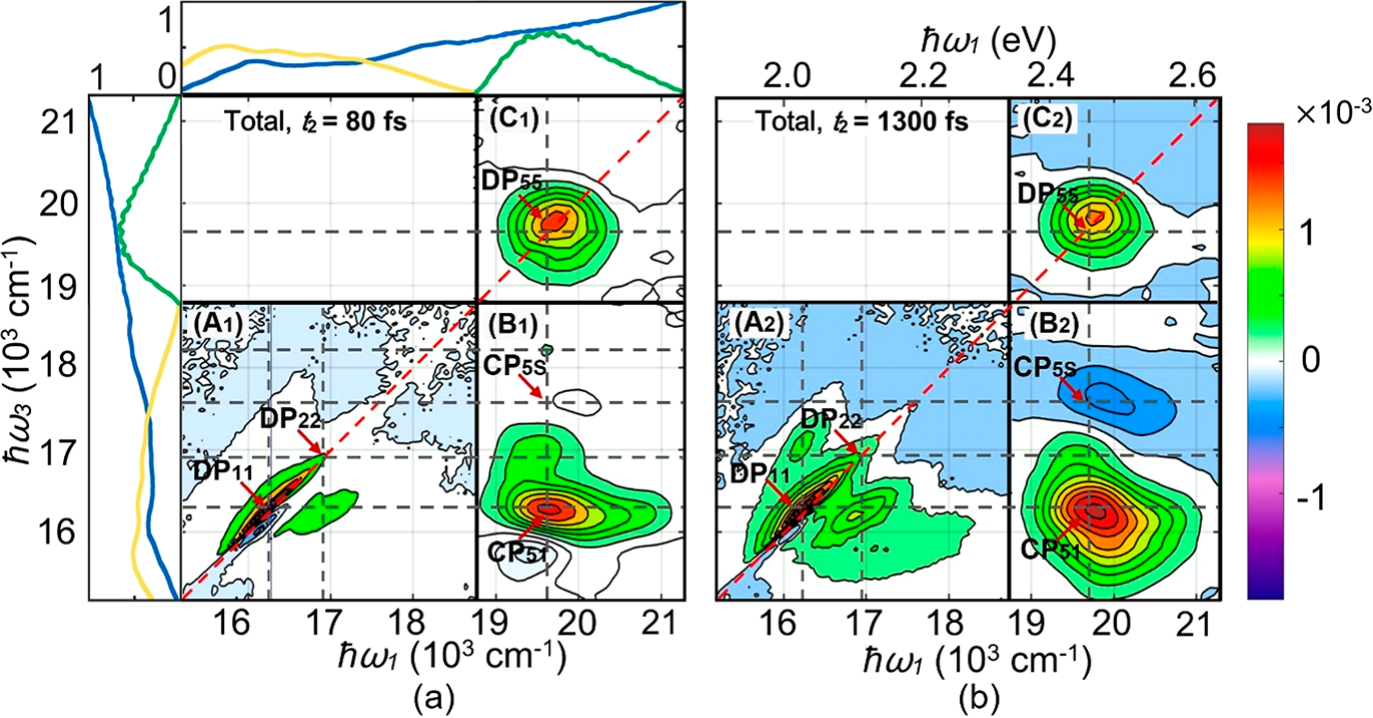
Real part of the total 2D spectroscopy signal at two population times, assembled from three separate single-color (A and C) and two-color (B) measurements. The black horizontal dashed lines mark the positions of the states |*e*_1_⟩, |*e*_2_⟩, |*e*_3_⟩, |*e*_4_⟩, and |*e*_5_⟩ at 16200, 16900, 17800, 18300, and 19700 cm^–1^, respectively. The three vertical lines mark the states which lead to the clear cross peaks. The labeled diagonal peaks (DP) and cross peaks (CP) are discussed further in the text. The leftmost and topmost panels show the laser spectra (yellow and green curves) and the absorbance of CdSe QDs at 77 K (blue curves).

CdSe QDs were prepared as in our previous work;^23^ for details see the Supporting Information.

The measured 2D signals can be divided into low- and high-energy regions. The wavenumber ranges of the low- and high-energy regions are from 15200 to 18800 cm^–1^ and from 18800 to 21300 cm^–1^, respectively. In the experimental results as shown in Figure 1, the *x*-axis refers to the excitation energy (ℏω_1_) and the *y*-axis corresponds the detection energy (ℏω_3_). The B_1_ and B_2_ panels are the two-color parts (the wavenumber range of the excitation energy is from 18800 to 21300 cm^–1^; the detection energy range is from 15200 to 18800 cm^–1^). The A_2_, B_2_, and C_2_ panels correspond to the population time *t*_2_ = 1300 fs. The red arrows point to the spectral features which are analyzed and discussed in detail. The labels DP and CP stand for diagonal peaks and cross peaks, respectively. To the left of and above the 2D plots, we show the absorption spectrum of the QDs (blue) and the pulse spectra (yellow and green).

We will discuss the three main diagonal peaks DP_11_, DP_22_, and DP_55_ and three of the cross peaks CP_52_, CP_51_, and CP_5S_. The subscript S represents the ESA signal possibly originating from a trap state as earlier discussed in ref (23), or it can be due to some other excited states. The two-color (off-diagonal) region contains the CP_52_, CP_51_, and CP_5S_ peaks. Among the above peaks, the DP_11_ and DP_22_ peaks were part of the analysis by Lenngren et al.^23^ showing hole trapping from the corresponding states.

Here, we mainly analyze the relaxation dynamics in the high-energy region. We use the total and rephasing parts of the 2D spectrum to illustrate the details of excited-state transitions of CdSe QDs.

The excited-state dynamics of CdSe QDs are analyzed based on the total and rephasing 2D maps measured at 77 K. The main excitonic states were identified based on Norris and Bawendi’s work,^40^ extending the analysis in Lenngren et al.^23^ The measured excitonic states are shown in Table 1. For more details of these fits, refer to the Supporting Information.

**Table 1:** Excited States of CdSe QDs in the 2D Electronic Spectrum

excitonic states	|*e*_1_⟩^23^	|*e*_2_⟩^23^	|*e*_3_⟩^23^	|*e*_4_⟩^23^	|*e*_5_⟩
symbols	1*S*_3/2_(*h*) – 1*S*(*e*)	2*S*_3/2_(*h*) – 1*S*(*e*)	1*S*_1/2_(*h*) – 1*S*(*e*)	1*P*_3/2_(*h*) – 1*P*(*e*)	2*S*_1/2_(*h*) – 1*S*(*e*)
energy (cm^–1^)	16200	16900	17800	18300	19700

We make use of the standard state nomenclature widely used for describing the excited states of QDs.^40^ The states are represented by a combination of the electron and hole principal quantum numbers and angular momentum states such as 1*S*, 1*P*, and 1*D*, together with the total angular momentum term for holes, which is 3/2 or 1/2 for the states discussed here.^41,40^ There are five excited states of CdSe QDs identified in the spectral coverage of our 2D spectroscopy experiment.

Let us take a closer look at the |*e*_5_⟩ state which originates from the 2*S*_1/2_(*h*) hole and the 1*S*(*e*) electron.^40^ The DP_55_ peak mainly originates from this state (see the spectral fit in the Supporting Information) with negligible contribution from higher energy transitions like 3*S*_1/2_(*h*) – 1*S*(*e*).^41,40^Figure 2 illustrates the possible transitions due to the above five excited states. To account for the ESA signal, we also need to consider the doubly excited manifold |*f*⟩. The |*f*⟩ manifold covers a wide range of transitions from the states |*e*_1_⟩, |*e*_2_⟩, |*e*_3_⟩, |*e*_4_⟩, and |*e*_5_⟩ and the possible other excited states like traps. The specific details are discussed in the following section and also shown in Figure 4.

**Figure 2:**
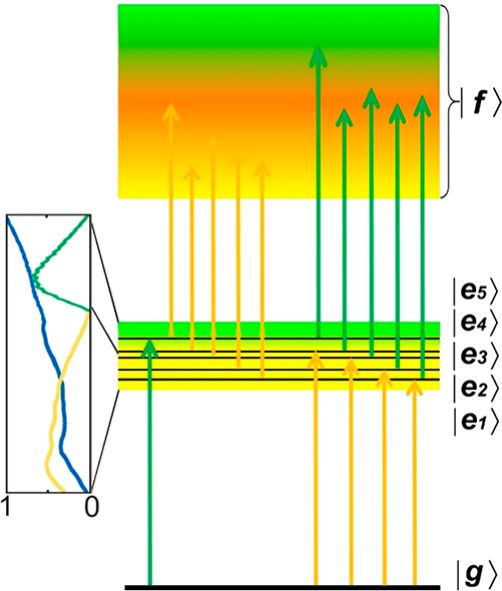
Singly and doubly excited energy levels together with the possible transitions that are driven by the pulses of two different energies. The yellow region is the low-energy area, and the green region is the high-energy area. In the left panel, the laser spectra are represented by yellow and green curves and the absorption of CdSe QDs as a blue curve. The yellow arrows indicate the transition processes due to the low-energy pulses. Similarly, the green arrows represent the possible transitions due to the high-energy pulses.

In Figure 3, the peak DP_11_ mainly comes from the excited state |*e*_1_⟩ at around 16200 cm^–1^. Elongation of the peak DP_11_ along the diagonal of the 2D map originates from inhomogeneous broadening due to the size distribution of CdSe QDs. To further understand the relaxation dynamics of CdSe QDs among |*e*_1_⟩, |*e*_5_⟩, and other excited states, we analyze the evolution of the 2D spectra during the population time from 0 to 1400 fs. The CP_51_, CP_52_, and CP_5S_ peaks, appearing in the lower right corner, and the DP_55_ peak, appearing in the upper right corner, are all related to the excitation of the state |*e*_5_⟩ at 19700 cm^–1^.^40^ The cross peaks CP_51_ and CP_52_ are visible already at early times (Figures 1a and 3a), showing the correlation of states |*e*_5_⟩, |*e*_2_⟩, and |*e*_1_⟩. The dynamics seen in the population time dependence of CP_51_, CP_5S_, and DP_55_ peaks in Figure 3 reflect the population relaxation through the ladder of the levels and represent the overall population dynamic from 21300 to 15500 cm^–1^. The structure at ℏω_1_ > 20500 cm^–1^ might be due to signals from higher-energy states or the dispersive line shape of the 2D spectra associated with |*e*_5_⟩.

**Figure 3:**
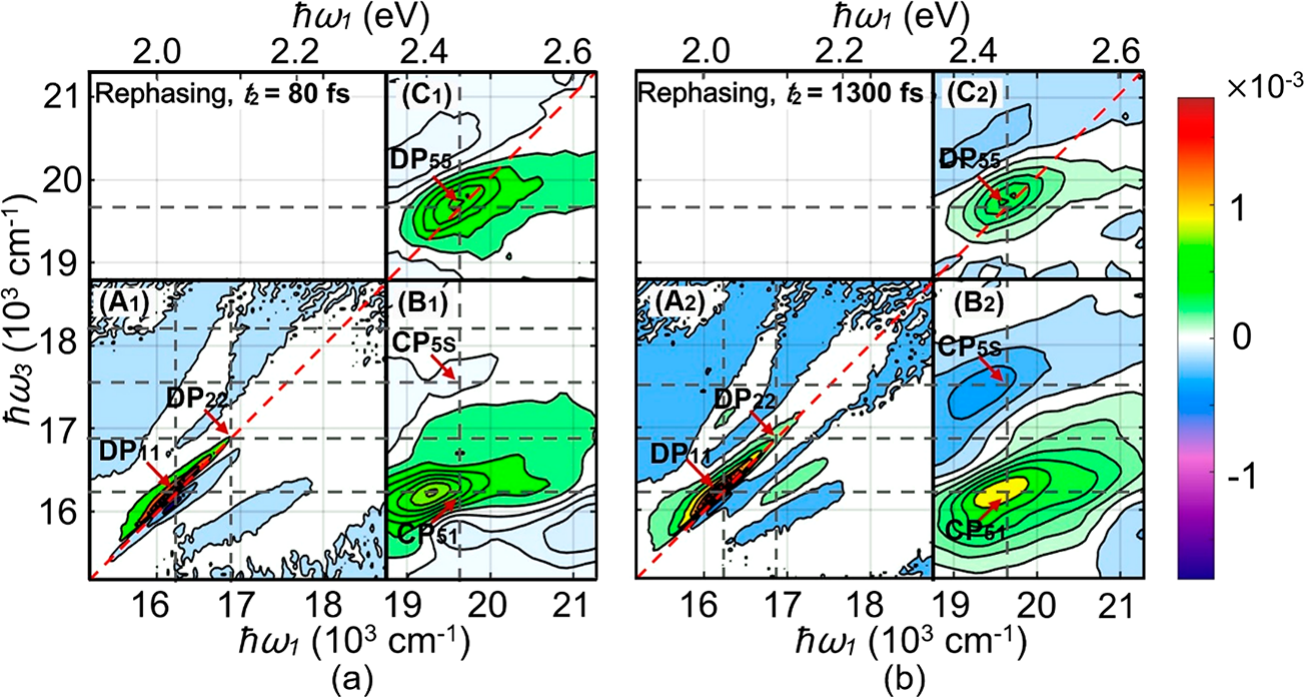
Real part of the rephasing 2D spectrum at two representative time points, assembled from two single-color (A, C) and a two-color (B) measurement.

We follow the peak changes until *t*_2_ = 1400 fs. After that, no further changes occur apart from the overall decay of the excited state.^23^ To avoid the possible nonresonant signal components during the pulse overlap (see Figure S6), we start the analyses from 80 fs. The population time dependence of the most significant features of the real part of the 2D spectra is shown in Figure 4. The cuts of the three 2D peaks taken at |*e*_5_⟩ excitation are shown in Figure 4a. The cross peak CP_51_ corresponds to the detection of |*e*_1_⟩ and DP_55_ to the detection of |*e*_5_⟩, while the origin of the ESA component CP_5S_ cannot be uniquely identified due to the uncertainty of the energy of the state responsible for ESA signals. The three kinetic traces in Figure 4b show the population time dependence of the peaks DP_55_, CP_5S_, and CP_51_, respectively. Each curve was fitted by an exponential function as shown in Figure 4b. The lifetimes obtained for these peaks are 100 ± 10, 700 ± 50, and 700 ± 50 fs, respectively. For more details, refer to the Supporting Information.

**Figure 4:**
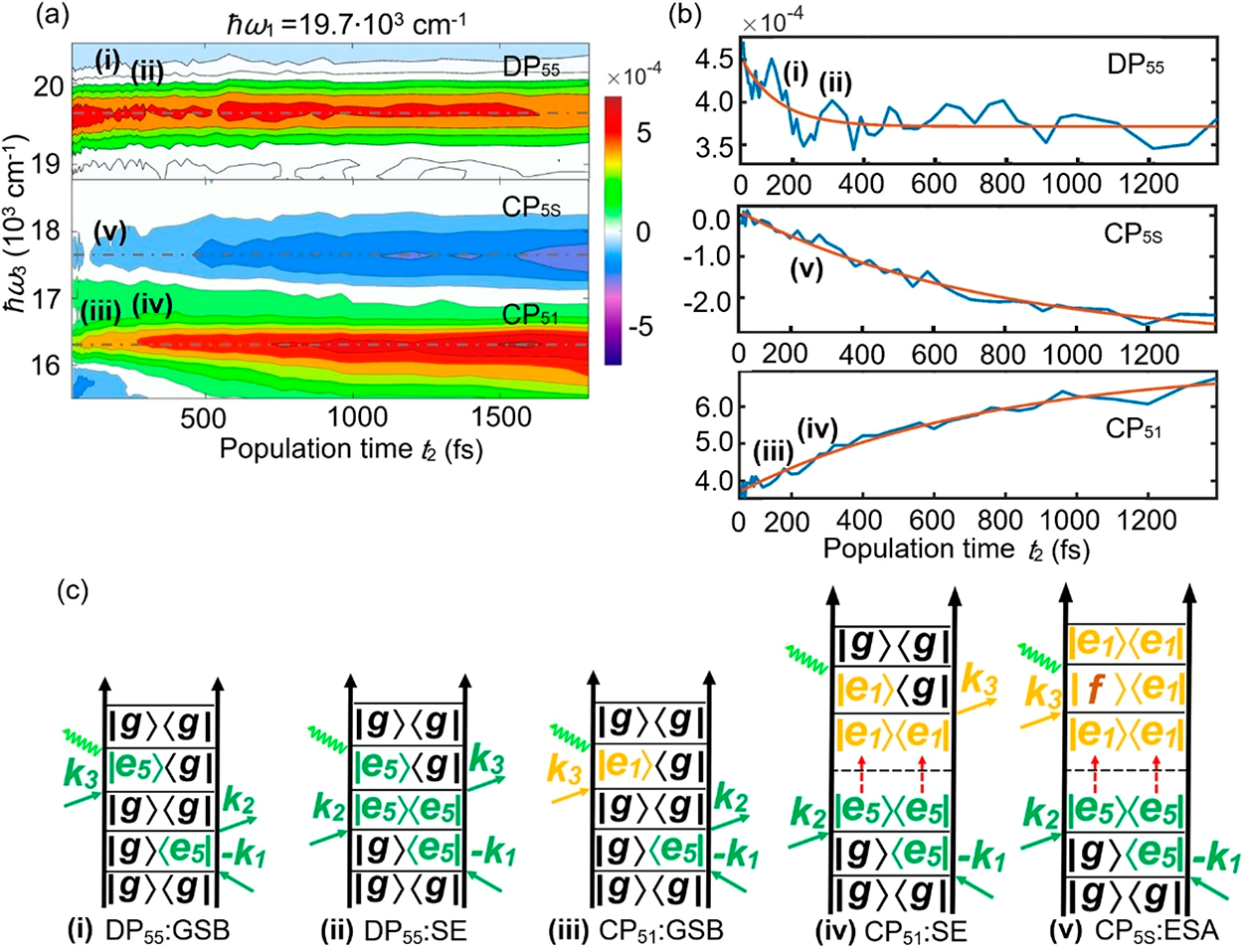
Population-time dependence of the main spectral features. (a) The real part of the three main peaks of the rephasing 2D spectra as a function of the population time *t*_2_. (The results without normalization can be found in the Supporting Information.) (b) Kinetics of the three main peaks DP_55_, CP_5S_, and CP_51_. The exponential decay and rise times of the three curves are 100 ± 10, 700 ± 50, and 700 ± 50 fs, respectively. (c). Feynman diagrams (i) and (ii) are the GSB and SE pathways for the DP_55_ peak. Feynman diagram (iii) represents the GSB pathway for the CP_51_ peak. Feynman diagram (v) indicates the relaxation processes of CP_5S_.

The decaying diagonal peak DP_55_ is well described by the GSB and SE pathways (see Figure 4c). The contribution of SE gradually decreases in DP_55_ since the population is relaxing. The 100 fs decay, therefore provides the relaxation time of the initially created |*e*_5_⟩. The contribution of GSB to DP_55_ is nearly constant within the measured population time; see the corresponding Feynman diagrams. The signal lives as long as the ground state is recovered. The growth of the band CP_5S_ corresponds to the arrival of the initial |*e*_5_⟩ population in a state which absorbs at about 17500 cm^–1^. The subscript S indicates that we cannot uniquely identify the state based on the ESA signal only. For example, in our earlier work,^23^ the analogous signal at lower energy excitation was assigned to a trap state. The band CP_51_ is mainly due to the GSB(iii) and SE(iv) pathways. While the GSB is constant, the SE grows with time. The corresponding time constant is 700 fs, telling us the time it takes for the |*e*_5_⟩ excitation to relax to |*e*_1_⟩. Because this is very similar to the growth of the CP_5S_, we conclude that both bands originate from the same state, also suggesting a reinterpretation of the ESA feature around ℏω_1_ = 18300 cm^–1^ and ℏω_3_ = 17500 cm^–1^ (labeled F in our previous work^23^).

At first glance, the time constants seem to contradict—we say that the relaxation from |*e*_5_⟩ takes 100 fs, but the arrival to |*e*_1_⟩ corresponds to a 700 fs time constant. To explain this, we need to consider the relaxation mechanism—coupling to the LO phonons with the frequency of about 200 cm^–1^ in CdSe. Because the electronic relaxation gives energy away to the nuclear lattice, this phonon quantum is the largest possible relaxation step. The higher-order multiphonon steps are significantly less likely to occur. The energy gap between |*e*_5_⟩ and |*e*_1_⟩ is 3400 cm^–1^. Thus, it takes over 15 jumps to relax from the |*e*_5_⟩ to the |*e*_1_⟩ state. This brings up another contradiction—the energy gap between the QD states is far larger than the phonon energy, suggesting a drastic slowdown of the relaxation. The expected reduction of the relaxation in QDs is called the phonon bottleneck. As explained by atomistic calculations^42^ of the QD electronic band structure, even though the spectral features follow the effective-mass theory nomenclature that we use, in reality, the QD electronic bands are quasi-continuous with a significant number of states to provide efficient phonon-induced relaxation pathways down to the band edge. When exciting into |*e*_5_⟩, we do not rule out hole trapping from |*e*_1_⟩ as described by our previous work^23^ as part of a multistep relaxation pathway, but it is not observable directly in the two-color data due to the strong CP_51_ peak.

The two-color 2D spectroscopy provides additional spectral coverage and thereby allows access to the relaxation dynamics from higher excited states in QDs. A diagonal peak (DP_55_) and three cross peaks (DP_11_, CP_51_, and CP_5S_) were observed by 2DES in this region. We clarified the relaxation dynamics in QDs based on these main 2D spectral bands. The relaxation occurs through the coupling to the LO phonons, and it takes multiple consecutive relaxation jumps to reach the low-energy band edge. The relaxation process takes about 700 fs.

## Supporting Information Available

Additional material explaining details of the sample preparation, 2DES setup and the experimental conditions, further experimental results, energy level system of CdSe QDs, and data processing (PDF)
