# Supplementary material for: Excited States and Their Dynamics in CdSe Quantum Dots Studied by Two-Color 2D Spectroscopy
Source: J Phys Chem Lett. 2022 Jan 28;13(5):1266–71. doi: 10.1021/acs.jpclett.1c04110 (PMC8842281; doi:10.1021/acs.jpclett.1c04110)
Supplement: Supplementary file 1 — jz1c04110_si_001.pdf [file jz1c04110_si_001.pdf]

Supporting Information

Excited States and Their Dynamics in CdSe Quantum Dots Studied by Two-Color 2D Spectroscopy

Zhengjun Wang,[†] Nils Lenngren,[†,‡] Edoardo Amarotti,[†] Albin Hedse,[†] Karel Žídek,[†,$] Kaibo Zheng,[†,#] Donatas Zigmantas,[†] Tõnu Pullerits[†]

[†] Division of Chemical Physics and NanoLund, Lund University, P.O. Box 124, 22100 Lund, Sweden.

[‡] ELI Beamlines, Institute of Physics, Czech Academy of Sciences v.v.i., Za Radnicí 835, 252 41 Dolní Břežany, Czech Republic.

[$] Regional Center for Special Optics and Optoelectronic Systems (TOPTEC), Institute of Plasma Physics, Czech Academy of Sciences v.v.i., 182 00 Prague 8, Czech Republic.

[#] Department of Chemistry, Technical University of Denmark, DK-2800 Kongens Lyngby, Denmark.

Contents

S1. Sample Preparation S2

S2. 2DES Setup and Conditions..S2

S3. Extension of Experimental Results S3

S4. Energy Level System of CdSe QDs S4

S5. Data Processing S4

S1. Sample Preparation

The sample of CdSe QDs analyzed in this paper has been prepared as described previously,[1,2,3] using 3-mercaptopropionic acid as capping agent and ethanol as solvent. The sample was then redispersed in a 1:1 mixture of methanol and ethanol and cooled to 77 K, forming a solvent glass.[1] The CdSe QDs sample has a mean diameter of about 7 nm. The size distribution of quantum dots is from 6.3 nm to 8 nm in diameters. The absorption spectra of the CdSe QDs sample are collected at room temperature (spectra not shown here) or at 77 K. Specific details are described in references.[1]

S2. 2DES Setup and Conditions

2DES spectroscopy was performed using the setup previously reported.[4] A 1030-nm pulse is generated in a Yb:KGW laser at 10 kHz and passed through a nonlinear optical parametric amplifier. The output is then compressed into a 9.2 fs long pulse (FWHM) with a diameter of 100 µm.

Three different spectral energy areas were measured. For the first set of measurements (the low-energy area), we used 1-nJ pulses centered at 600 nm, with photon wavenumbers ranging from 15200 cm^{-1} to 18800 cm^{-1}. For the second set (the high-energy area), the pulse energy was 2 nJ

and the central wavelength was 500 nm, with photon wavenumbers from 18800 cm^{-1} to 21300 cm^{-1}. The third set (the two-color measurement) was measured with the first two pulses as in the high-energy area (2 nJ, 500 nm, 15200 cm^{-1} to 18800 cm^{-1}) and the third pulse as in the low-energy area (1 nJ, 600 nm, 18800 cm^{-1} to 21300 cm^{-1}). A delay stage (for population time) and a glass wedge (for coherence time) are used to delay the pulses. The signal beam is detected by heterodyne detection in the phase-matching direction. An interferogram is recorded using a CCD coupled to the spectrometer.

S3. Extension of Experimental Results

The states in the low-energy region were identified in our previous work[1] as recapitulated in **Figure S1**. According to Norris and Bawendi[5] and our fit shown in **Figure S2**, there are two states in the high-energy region: the strong state $2S_{1/2} - 1S(e)$ at 19700 cm^{-1} and the weak state $3S_{1/2} - 1S(e)$ at 20600 cm^{-1}. In our experimental data, e_5 plays a leading role in contribution comparing with the other states, and we assign it as $2S_{1/2} - 1S(e)$.

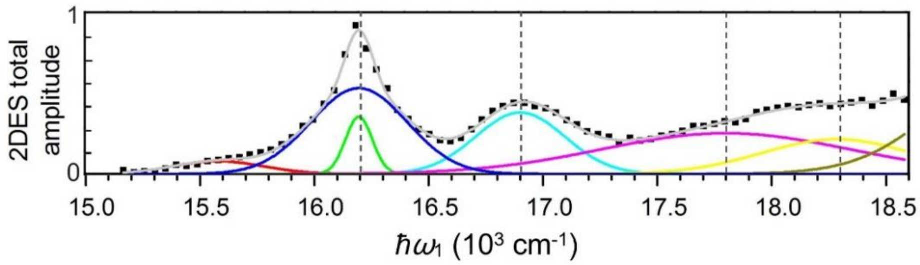

Figure S1. Fitting in the low-energy region of the excited states of CdSe QDs from the amplitude part of the total 2D spectrum at t_2 = 10 ps and $\hbar\omega_3$ = 16 200 cm^{-1}, divided by the laser spectrum, as shown in our previous work[1]. The data (black squares) are fitted as a sum (gray line) of Gaussians corresponding to specific states (colored lines, average energies are marked by vertical dashed lines).

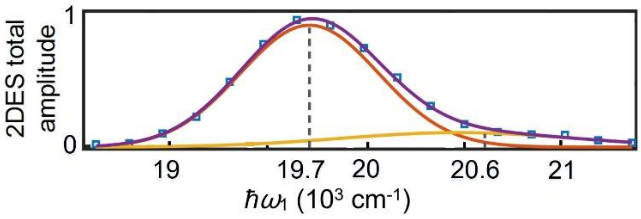

Figure S2. Fitting in the high-energy region of the excited states of CdSe QDs from the amplitude part of the total 2D spectrum at t_2 = 10 ps along the diagonal, divided by the laser spectrum. This time, we fit along the diagonal of the 2D spectrum, since the diagonal peaks correspond to linear absorption positions.[6] The purple line is the sum of the two fitted Gaussians (red and yellow, with average energies marked by vertical dashed lines), and the blue squares are the experimental data.

S4. Energy Level System of CdSe QDs

Excitons of CdSe QDs[1,5] are combinations of electrons and holes, as shown in **Figure S3**.

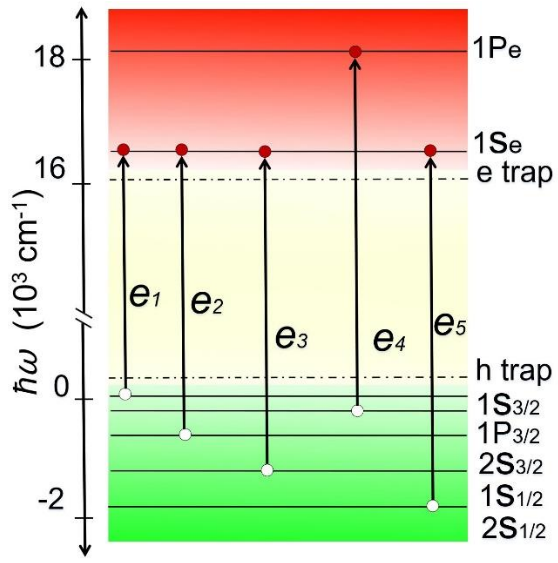

Figure S3. The excited states e_1, e_2, e_3, e_4, and e_5 in CdSe QDs.

We use the following labels e_1, e_2, e_3, e_4 and e_5 to represent the excited states $1S_{3/2} - 1Se$, $2S_{3/2} - 1Se$, $1S_{1/2} - 1Se$, $1P_{3/2} - 1Pe$, and $2S_{1/2} - 1Se$, respectively.

S5. Data Processing

The 2D spectrum in **Figure S4**(b) is obtained from the 2D slice of the 3D data in **Figure S4**(a) at $\hbar\omega_1$ = 19700 cm^{-1}, among the excited states $|e_1\rangle$, $|e_5\rangle$, and other excited states. The data in **Figure S4**(c) are the kinetics of DP_{55}, CP_{5S}, and CP_{51}, respectively, which are the features at $\hbar\omega_1$ = 19700 cm^{-1}, 17500 cm^{-1}, and 16200 cm^{-1}.

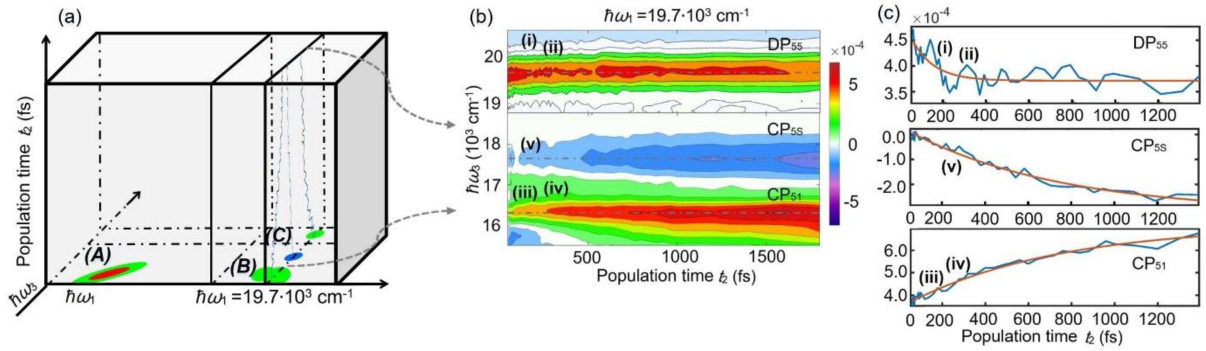

Figure S4. Schematic diagram of data processing for the real part of the rephasing 2D spectrum.

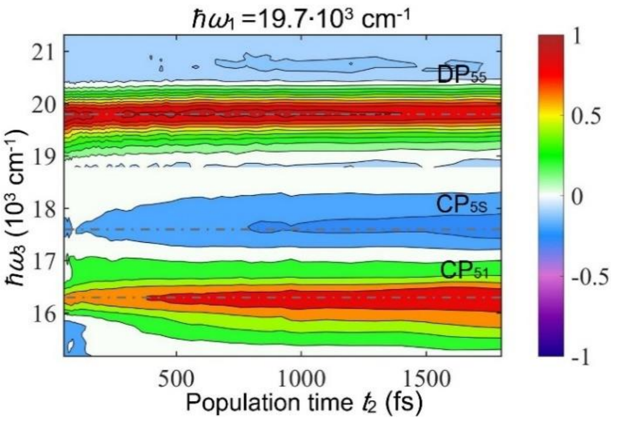

Figure S5. The real part of the total 2D spectrum using the same normalization for all panels.

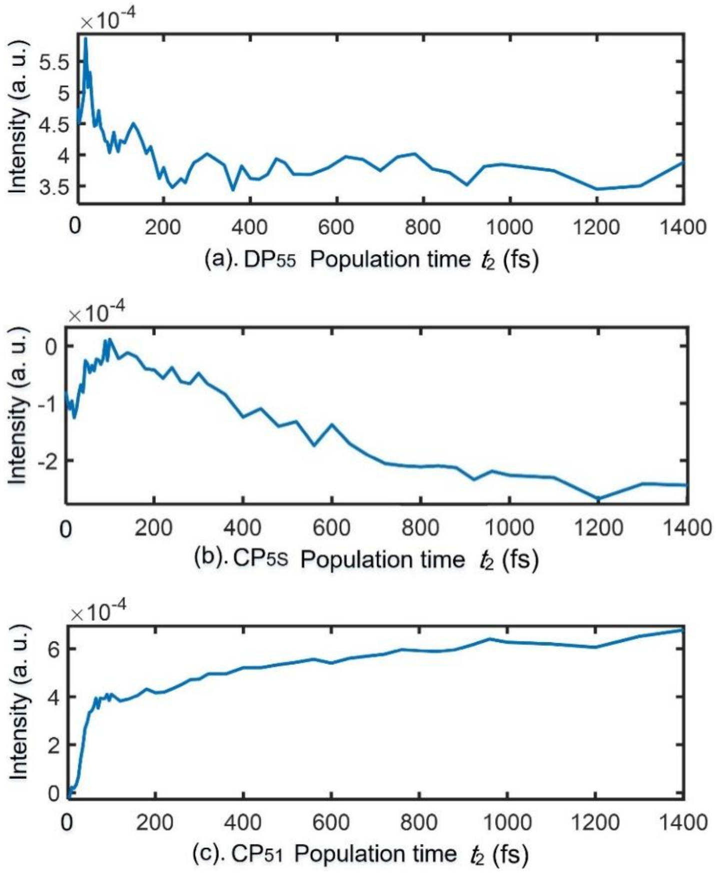

Figure S6. The intensities of DP_{55}, CP_{5S}, and CP_{51} from the real part of the rephasing 2D spectrum.

In addition, a summary of the different population times for the detection of the three panels in **Figures 1** and **3** of the main text is shown in **Table S1**.

Table S1. The panels of 2D spectra

Population time t_2	Single-color	Two-color	Single-color
t_2=80 fs	A_1	B_1	C_1
t_2=130 fs	A_2	B_2	C_2

References

(1) Lenngren, N.; Abdellah, M. A.; Zheng, K.; Al-Marri, M. J.; Zigmantas, D.; Žídek, K.; Pullerits, T. Hot Electron and Hole Dynamics in Thiol-Capped CdSe Quantum Dots Revealed by 2D Electronic Spectroscopy. *Phys. Chem. Chem. Phys.* **2016**, *18* (37), 26199–26204.

(2) Bullen, C. R.; Mulvaney, P. Nucleation and Growth Kinetics of CdSe Nanocrystals in Octadecene. *Nano Lett.* **2004**, *4* (12), 2303–2307.

(3) Zheng, K.; Žídek, K.; Abdellah, M.; Zhang, W.; Chábera, P.; Lenngren, N.; Yartsev, A.; Pullerits, T. Ultrafast Charge Transfer from CdSe Quantum Dots to P-Type NiO: Hole Injection vs Hole Trapping. *J. Phys. Chem. C.* **2014**, *118* (32), 18462–18471.

(4) Augulis, R.; Zigmantas, D. Two-Dimensional Electronic Spectroscopy with Double Modulation Lock-in Detection: Enhancement of Sensitivity and Noise Resistance. *Opt. Express.* **2011**, *19* (14), 13126-13133.

(5) Norris, D.; Bawendi, M. Measurement and Assignment of the Size-Dependent Optical Spectrum in CdSe Quantum Dots. *Phys. Rev. B - Condens. Matter Mater. Phys.* **1996**, *53* (24), 16338–16346.

(6) Brixner, T.; Stenger, J.; Vaswani, H. M.; Cho, M.; Blankenship, R. E.; Fleming, G. R. Two-Dimensional Spectroscopy of Electronic Couplings in Photosynthesis. *Nature.* **2005**, *434* (7033), 625–628.
